# Fibronectin and Its Receptors in Hematopoiesis

**DOI:** 10.3390/cells9122717

**Published:** 2020-12-18

**Authors:** Franziska Wirth, Alexander Lubosch, Stefan Hamelmann, Inaam A. Nakchbandi

**Affiliations:** 1 Institute of Immunology, University of Heidelberg, 69120 Heidelberg, Germany; franziska.wirth@immu.uni-heidelberg.de (F.W.); alexander.lubosch@immu.uni-heidelberg.de (A.L.); stefan.hamelmann@immu.uni-heidelberg.de (S.H.); 2 Max-Planck Institute for Medical Research, 69120 Heidelberg, Germany

**Keywords:** fibronectin, hematopoiesis, integrin, adhesion, migration, homing, myelopoiesis, thrombopoiesis

## Abstract

Fibronectin is a ubiquitous extracellular matrix protein that is produced by many cell types in the bone marrow and distributed throughout it. Cells of the stem cell niche produce the various isoforms of this protein. Fibronectin not only provides the cells a scaffold to bind to, but it also modulates their behavior by binding to receptors on the adjacent hematopoietic stem cells and stromal cells. These receptors, which include integrins such as α4β1, α9β1, α4β7, α5β1, αvβ3, Toll-like receptor-4 (TLR-4), and CD44, are found on the hematopoietic stem cell. Because the knockout of fibronectin is lethal during embryonal development and because fibronectin is produced by almost all cell types in mammals, the study of its role in hematopoiesis is difficult. Nevertheless, strong and direct evidence exists for its stimulation of myelopoiesis and thrombopoiesis using in vivo models. Other reviewed effects can be deduced from the study of fibronectin receptors, which showed their activation modifies the behavior of hematopoietic stem cells. Erythropoiesis was only stimulated under hemolytic stress, and mostly late stages of lymphocytic differentiation were modulated. Because fibronectin is ubiquitously expressed, these interactions in health and disease need to be taken into account whenever any molecule is evaluated in hematopoiesis.

## 1. Introduction

The bone marrow represents a unique environment optimized for hematopoiesis. On one hand, hematopoietic stem cells in the marrow need to be preserved throughout life, and on the other hand, they need to respond to acute changes in the requirement for various types of blood cells. They do this by answering cues from their surroundings, the neighboring cells, or distant organs. A variety of cells are located in close proximity of the stem cells, providing them with a protective environment, but also with short-distance signals to allow them to function. This niche was initially thought to be close to the bone lining cells or osteoblasts [1], but later work seems to localize the long-term hematopoietic stem cells in proximity to endothelial cells [2,3]. All these cells are embedded in a meshwork of extracellular matrix proteins produced by the supportive cells. This matrix modifies stem cell behavior by acting on receptors located on the surface of the stem cells and the supporting cells. Since matrix consists of a large number of molecules, and each affect different receptors, the matrix can exert a panoply of effects. Lastly, the matrix serves as a reservoir for growth factors changing their availability depending on its composition [4].

## 2. The Rationale for Evaluating Fibronectin and Its Receptors

Several extracellular matrix proteins have been examined in the context of hematopoiesis [5,6,7], but fibronectin stands out for a variety of reasons. It is produced by most mammalian cells. In the bone marrow, almost all cell types produce fibronectin, such as endothelial cells, pericytes, and osteoblasts [8,9,10,11,12,13]. Furthermore, circulating fibronectin infiltrates various tissues, including bone [14,15]. It can, therefore, be viewed as an integral component of the stem cell niche.

Fibronectin is a cell adhesion molecule. It consists of a heterodimer of two amino acid chains. These chains are composed of structural units lined up next to each other that can be classified into three types, type I, II, and III. Most units are similar between both chains, but three domains may differ: The extracellular domain A (EDA), the extracellular domain B (EDB), and the variable region (V). The isoform called plasma fibronectin lacks both EDA and EDB domains. While EDA or EDB can either be present or absent, the variable region can undergo alternative splicing [16]. Lastly, post-translational modifications further modulate the interaction of the molecule with the cells [17]. Fibronectin exerts a variety of functions by acting on cell surface receptors. It modulates proliferation, inhibits apoptosis, supports migration, and regulates differentiation [9,11,12]. It is, therefore, not surprising that fibronectin deletion during embryonic life is lethal [18]. Deletion in adult life using the Cre-LoxP system helped shed light on various postnatal roles [19]. This large variety of effects is possible through the presence of the various isoforms and post-translational modifications, as well as the large number of receptors to which fibronectin can bind.

## 3. Fibronectin Receptors Relevant for Hematopoiesis

Fibronectin mostly binds to integrins, which consist of heterodimers of an α and a β subunit located on the cell surface. Integrins not only bind matrix and affect intracellular changes, but processes in the cell can themselves change the activation state of integrins, making them more likely to react to their surroundings [20]. Of the integrins able to bind fibronectin, five have been evaluated in hematopoiesis: α5β1 [21], α4β1 [22], α4β7 [23], α9β1 [24], and αvβ3 [25], all of which are expressed on hematopoietic stem cells (Table 1).

The classical fibronectin receptor is α5β1, which binds to the RGD (Arginine-Glycine-Aspartate) sequence in fibronectin. A site called connecting segment 1 (CS1) located in the variable region can bind α4β1 and α4β7 integrin. However, two more sites called H1 and CS5 offer binding sites to α4β1 integrin [16]. If CS1 is O-glycosylated at threonine 33, binding to α4β1 is affected by suppressing signaling through this integrin [17]. Interestingly, the presence of the EDA domain alters the characteristics of the binding to the classical fibronectin receptor α5β1 [19,29], but EDA has itself a binding site to two other integrins α4β1 and α9β1 [30,31]. A third integrin called αvβ3 binds through the RGD sequence. This binding is modified by the presence of the EDB domain leading to increased intracellular response [32,33]. Close to the N-terminal of fibronectin, a site was found that binds to α5β1 with low affinity and αvβ3 with high affinity [34]. Fibronectin can also engage other integrins that will not be mentioned here (reviewed in [16]).

Three non-integrin receptors can bind to fibronectin. These are: Toll-like receptor-4 (TLR-4), CD44, and syndecan-4. Toll-like receptors (TLRs), including TLR-4 are expressed on hematopoietic stem cells [26,27], as is CD44 [28], but not syndecan-4.

Toll-like receptors are members of the receptors that recognize bacterial molecular patterns (also called pathogen-associated molecular patterns, PAMPs) located on immune cells, as well as other cell types. TLR-4 normally recognizes lipopolysaccharide (LPS) on gram-negative bacteria or lipoteichoic acid on gram-positive bacteria and induces the cells to produce cytokines, chemokines, and antibacterial/antiviral peptides. Both the EDA domain or the type III-1 domain can activate TLR-4 [35,36]. At first, it may seem surprising that fibronectin, which circulates in the bloodstream, would activate a proinflammatory receptor [37]. Most studies, however, used a fragment containing EDA or III-1 and not the whole molecule. It is, therefore, conceivable that the effect of EDA-containing fibronectin on TLR-4 is limited. Furthermore, fibronectin itself can be viewed as an opsonizer that facilitates phagocytosis of bacteria [32].

CD44 is considered a hyaluronic acid receptor, because it contains a hyaluronic acid-binding pouch. The binding affinity to hyaluronic acid, however, is affected by various post-translational modifications. The presence of chondroitin sulfate on one of the isoforms of CD44 allows it to bind fibronectin [38]. Of note, α4β1 and CD44 were found to cooperate to bind stromal cells to fibronectin [39].

Syndecan-4 is a proteoglycan that spans the cell membrane and can bind to fibronectin [40]. It modifies the expression of integrin pairs at the cell surface: If it is Src phosphorylated, it enhances α5β1 degradation and increases αvβ3 [41]. It is not expressed on hematopoietic stem cells, but can be detected on monocytes and lymphocytes [42,43].

## 4. Steps of Hematopoiesis

During development, hematopoiesis starts early in the yolk sac, then moves to the liver and later to the bone marrow. This requires the migration of stem cells to the liver and then to the bone marrow. Once in the liver or the bone marrow, the stem cells need to initiate an interaction with the stromal cells and the matrix to be retained in the niche. Afterward, the stem cells may proliferate, giving rise either to a self-renewed stem cell or to a cell that starts differentiating to form the various blood cell types [44].

Once differentiation is initiated, the hematopoietic stem cell gives rise to the multipotent progenitor cells (MPPs). The stem cells and the multipotent progenitor cells together constitute the population characterized by the expression of c-Kit and Sca-1 in the absence of lineage markers for differentiated blood cells (L^−^S^+^K^+^: Lineage-negative_Sca-1-positive_c-Kit-positive). These two groups are also known as the hematopoietic stem and progenitor cells (HSPC). For brevity in this review, the word stem cells refer to this HSPC population, since most papers did not characterize this population further or used variable sets of markers. The MPPs differentiate further to become either a common myeloid progenitor (CMP) or a common lymphoid progenitor (CLP). CMPs give rise to megakaryocyte-erythrocyte progenitors (MEPs) or granulocyte-monocyte progenitors (GMPs), while CLPs give rise to B and T-cells. NK cells also originate from CLPs, while dendritic cells can originate from either CMPs or CLPs. Platelets and red blood cells originate from MEPs, while various monocytic population, macrophages, and granulocytes from GMPs and lymphocytes from CLPs [45].

## 5. Fibronectin and Fibronectin Receptors in Hematopoietic Stem Cells

Because fibronectin is ubiquitously expressed in the bone marrow, characterization of the specific effects of fibronectin on hematopoietic stem cells has proven difficult [9,15,19,32]. Most of our knowledge on how fibronectin affects hematopoiesis is deduced from what was learned by evaluating the role of the various receptors, because fibronectin was not directly examined together with each of the receptors mentioned above. Since, however, fibronectin is present in abundance and can bind to these receptors, it is likely that fibronectin acts via these receptors.

In the following, we present first the evidence of the role of integrins in hematopoiesis followed by a discussion of the effects of fibronectin and fibronectin-binding receptors on stem cell behavior: Migration and homing, attachment and retention, proliferation, and finally, differentiation. There is some overlap between these various steps, however.

### 5.1. Evidence for A Role of Integrins in Hematopoiesis

The role of integrins can be inferred from evaluating the effect of deletion of kindlin-3, a molecule required for integrin activation and signaling. Kindlin-3 is expressed in hematopoietic cells, and knockout cells were detected in the fetal liver, suggesting that homing to the fetal liver is not kindlin-3 dependent [46]. This, however, does not fully exclude a role for integrins, because enough functionality of the integrins might be maintained to support this step despite the loss of kindlin-3.

Homing to the bone marrow is markedly diminished, but not completely lost in kindlin-3 knockout mice, since homing and retention of stem cells in the bone marrow, at least in part, rely on CXCL12/SDF-1α and its receptor CXCR4 [47,48]. Retention of quiescent cells was not affected in kindlin-3 knockout mice. Under stress conditions, however, homing to (and retention of) hematopoietic stem cells in the bone marrow requires the presence of functional integrins because kindlin-3 deletion depleted the hematopoietic stem cells [46]. A possible explanation is that signaling downstream of CXCR4/CXCL12 can activate integrins or that CXCL12 cooperates with integrins as was shown for homing and transmigration through the endothelium [21,49].

Dissecting the role of fibronectin in hematopoiesis is difficult, because of the presence of several fibronectin isoforms, each acting on different receptors. Adding to the complexity is that the receptors can have different effects depending on the developmental stage of the organism.

### 5.2. Migration and Homing

Fibronectin helps maintain the ability of the stem cells to reconstitute the bone marrow and supports engraftment [50]. This is mediated by fibronectin-binding to α5β1 integrin, possibly in cooperation with α4β1 and CD44 (Table 2).

Loss of β1 integrin in hematopoietic stem cells prevents migration to the fetal liver or engraftment in the bone marrow of irradiated recipient adult mice. This underscores the importance of β1 integrins in homing. In comparison, even though CXCL12/SDF-1 is a strong chemoattractant of hematopoietic stem cells and an important molecule for migration of stem cells to the bone marrow, deletion of its receptor markedly diminishes, but does not fully abrogate engraftment of hematopoietic stem cells in adult mice [47,51].

Of the integrins that require β1 subunit, the fibronectin receptor α5β1 supports the homing of progenitor cells to the bone marrow, as evidenced by decreased homing with the use of an inhibitory antibody [21]. α4 is another α subunit that pairs with β1. Using chimeric mice (in which stem cells from knockout mice are transplanted into wildtype mice), it was determined that α4 is not required during embryonic development for migration of hematopoietic progenitors [52]. In adult mice, the use of inhibitory antibodies against α4β1 or knockout of α4 suggested α4 is essential for homing to the bone marrow after irradiation and transplantation [53,54,55].

In human hematopoietic stem cells, an isoform of CD44 called HCELL (Hematopoietic Cell E-/L-selectin Ligand) is detected [38]. Its expression in human stromal cells led to homing to the bone marrow, even though CXCR4 was not expressed in these cells [56]. This isoform, however, is only found on human hematopoietic stem cells and not on murine stem cells. In mice, CD44 also supports homing of hematopoietic stem cells [28]. Since the engagement of CD44 activates α4β1, allows binding to fibronectin [39], and triggers cell movement in the absence of chemokines [38], it is possible that through the cooperation of CD44/α4β1 signaling on stem cells, their anchoring on mesenchymal stromal cells in the hematopoietic niche is enhanced.

### 5.3. Attachment and Retention

The stem cells are retained in the niche by adhering to stromal cells or to the matrix in the niche. If adherence is lost, the cells egress from the bone marrow and/or differentiate. Inhibiting the engagement of stem cells with their surrounding diminished the expression of stem cells-associated transcription factors cMyb and GATA-2 at the expense of increased expression of markers of differentiation [61]. This is compatible with the notion that the niche modifies the behavior of the stem cells depending on its composition.

**Figure 1:**
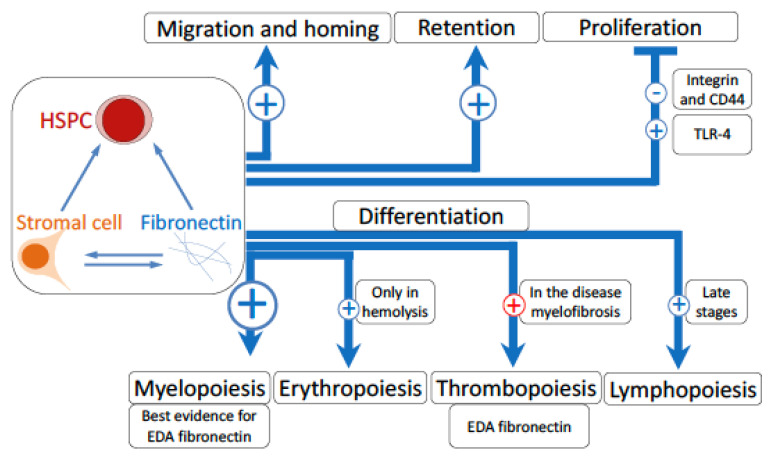
Binding to fibronectin and stromal cells supports various functions of the hematopoietic stem and progenitor cells (HSPC). Migration and homing, as well as retention are stimulated by fibronectin and/or integrins that bind fibronectin. Even though fibronectin normally stimulates cell proliferation, HSPC proliferation is inhibited when integrins engage with fibronectin. CD44 similarly inhibits proliferation. Once stimulated, the receptor TLR-4 enhances proliferation. The lower three arrows refer to the steps of differentiation. Myelopoiesis is increased by the EDA isoform of fibronectin. Erythropoiesis is only defective when fibronectin-binding integrins are manipulated in hemolytic anemia. Defects in thrombopoiesis are associated with fibronectin accumulation in the bone marrow in the disease called myelofibrosis. Defects in the late stages of lymphoid differentiation are seen in modulations of receptors able to bind fibronectin, but no direct link was established with fibronectin. The size of the circle coincides with the strength of the association with fibronectin. The red circle refers to a pathologic condition.

The ability of fibronectin to support hematopoietic stem cell attachment is so well established that it is used in assays analyzing adhesion of hematopoietic stem and progenitor cells in vitro [57]. The ability of stem cells to engraft in vivo is markedly improved if stromal cells are provided [62]. Since adding fibronectin has the same effect [63], it could be that stromal cells provide fibronectin for the stem cells to tether to it (Figure 1).

In vivo studies thoroughly evaluated the retention of stem cells in the niche. Neither deletion of β1 integrin in hematopoietic cells in the bone marrow in transplanted, and hence, chimeric adult mice, nor combined deletion of β1 and β7 in chimeric mice affected retention of hematopoietic stem cells in the bone marrow durably [23]. The application of antibodies against α4β1 integrin, however, mobilized stem/progenitor cells out of the niche, suggesting that α4β1 integrin contributes to the retention of the stem cells in the niche. In line with this, induced deletion of α4 in hematopoietic and several other cell types using Mx to drive Cre recombinase expression in floxed mice increased the release of progenitors into the peripheral blood [55].

The seemingly contradictory findings between β1 deletion and α4β1 modulation can be explained as follows: The experiments in which β1 and β7 chimera were produced affect the hematopoietic stem cells themselves [23,64], while the experiments with the α4 antibody and α4 deletion using Mx-Cre inhibit or diminish α4 on the stem cells and on cellular components of the niche unrelated to hematopoietic cells [55,58]. The various results, therefore, support the conclusion that deletion of α4 in surrounding cells changes their ability to support hematopoietic stem cells and prevent them from retaining the hematopoietic stem cells in the bone marrow, while α4 in the hematopoietic stem cells plays a limited role in their retention in the bone marrow.

One more integrin was evaluated, namely, αvβ3. Maintenance of long-term repopulating capacity of hematopoietic stem cells kept in culture or in vivo was found to be dependent on activation with thrombopoietin followed by αvβ3 [25]. While activation of β3 integrin was accomplished using vitronectin, because αvβ3 is an integrin for vitronectin, fibronectin is also able to bind to αvβ3, especially when the EDB domain is present [32]. Since osteoblasts produce EDB fibronectin, it is possible that this isoform originating from osteoblasts supports the maintenance of hematopoietic stem cells [12]. This has not been evaluated yet, however.

Taken together, these data suggest that fibronectin at least contributes to retaining the hematopoietic stem cells in their niche either directly by acting on αvβ3 or indirectly by acting on the supporting stromal cells.

### 5.4. Proliferation

Fibronectin usually stimulates cell proliferation [9]. In the absence of αv in hematopoietic cells (using Vav-iCre to delete αv in floxed mice) or periostin, the proliferation of hematopoietic stem cells increased, suggesting that αv engagement suppresses stem cell proliferation as is the case with α4β1 [59]. Signaling through β3 was found to maintain the long-term repopulation ability of stem cells in vivo [25]. Of note is that αvβ3 integrin requires several players that act in concert to modulate hematopoietic stem cells. Thrombopoietin, which binds to αvβ3, sets the stage via inside-out signaling to allow integrin binding to the extracellular matrix. This raises the possibility that fibronectin interaction with αvβ3 integrin affects proliferation indirectly.

As mentioned, TLR-4 is expressed in hematopoietic stem cells [26,27]. It can bind EDA-fibronectin and the domain III-1 [35,36] and is activated by surface molecules on bacteria. Thus, TLR-4 mediates an immune response against bacterial pathogens by enhancing the formation of myeloid cells, and hence, myelopoiesis in the case of infections [65]. This effect seems to be at least in part, indirect originating from cells other than the hematopoietic stem cells [66,67]. The activation of TLR-4 on hematopoietic stem cells increases proliferation [60], diminishes self-renewal, while inducing differentiation towards myelopoiesis [27]. In its absence, hematopoietic stem cell expansion is reduced, and the repopulating capacity is improved [68]. Since EDA-fibronectin stimulates TLR-4 signaling [69], it seems reasonable to assume that EDA-fibronectin affects hematopoiesis by enhancing proliferation.

Lastly, CD44 not only supports homing, but also negatively regulates proliferation [28].

### 5.5. Differentiation

The differentiation of hematopoietic stem cells is tightly regulated, leading to the production of the various cell types as needed by the organism. If a person bleeds, erythropoiesis is stimulated. Similarly, infections are associated with an increase in white blood cells that is partially due to an increase in the production of the needed cells in the bone marrow [70]. Our understanding of the mechanisms regulating how and why a hematopoietic stem cell moves along a certain differentiation path remains incomplete.

The role of fibronectin has been evaluated for myelopoiesis and thrombopoiesis. For the remaining hematopoietic steps, we will discuss the receptors that can bind to fibronectin, and at the same time, were studied in hematopoiesis (Table 3).

#### 5.5.1. Myelopoiesis

EDA-fibronectin increases myeloid differentiation of hematopoietic stem cells by binding to α5β1 integrin. This engagement modifies the response of the myeloid cell to cancer. As part of the study of the role of the osteoblastic niche in hematopoiesis, deletion of fibronectin in differentiating osteoblasts showed that myeloid cells of the bone marrow that differentiated in the absence of EDA were able to diminish cancer growth [19]. While the osteoblastic niche is thought of as consisting of bone lining cells, the collagen promoter used to drive Cre expression, and deletion of fibronectin in this model is only effective in differentiating osteoblasts [11]. This suggests that osteoblasts at various stages can modulate hematopoiesis. Since the addition of EDA-fibronectin enhanced myeloid differentiation, it appears that the niche can change hematopoietic cell behavior through the production of different components of the extracellular matrix.

The importance of which receptor is bound to fibronectin is highlighted by the disease of chronic myelogenous leukemia (CML). In this condition, an increase in dysfunctional myeloid cells is seen. Distorted adhesion by α4β1 and α5β1, increased survival of myeloid progenitors, and absence of adhesion to stromal cells leads to the uncontrolled increase in myeloid cells and disease manifestation [71]. Of note, however, is that physiologic myelopoiesis proceeds inefficiently in the absence of α4, but is not affected by the absence of α5 or αv [52,72]. The same is true for the deletion of β1 and β7 integrin subunits [23].

Bacteria, a major source of infections, can activate Toll-like receptors, including TLR-4, which is expressed on hematopoietic stem cells. This leads to the marked production of various cytokines pushing hematopoiesis towards the production of myeloid cells [66,70,73,74]. EDA or type III-1 domains in fibronectin were shown to bind to TLR-4, and hence, stimulation of TLR-4 by fibronectin would presumably be associated with an increase in myelopoiesis [36]. 

Finally, deletion of CD44, another fibronectin-binding receptor, increases monocytes in the bone marrow and peripheral blood, suggesting it is involved in regulating their differentiation [28].

#### 5.5.2. Erythropoiesis

In vitro experiments suggest that fibronectin modulates erythropoiesis [75,76], and induces apoptosis in hematopoietic malignancy [85]. In vivo studies produced complementary results. Combined β1 and β7 deletion in hematopoietic cells in chimeric mice showed limited recovery after induced hemolytic anemia [23]. In adult mice, deletion of α4 using the Mx promoter, which drives Cre expression in hematopoietic and non-hematopoietic cells, failed to show a difference in erythroid cells (Ter119^+^) in the bone marrow. After inducing acute hemolytic anemia, however, fewer reticulocytes and lower numbers of red blood cells were detected, suggesting a defect in erythropoiesis under stress [55]. Thus, α4β1 and/or α4β7 improve erythropoiesis after hemolysis.

Another set of experiments in vivo is also insightful. Deletion of integrins in erythroid cells using mice expressing the erythropoietin-receptor driving Cre expression in α4 or α5 floxed mice showed that α5 was not required for normal erythropoiesis, because even after stress, the mice lacking α5 in erythroid cells were similar to controls [77]. Deletion of α4 in erythrocytes both early or late during erythroid differentiation led to the retention of erythroblasts in the bone marrow and hampered the last steps in erythrocyte differentiation.

Taken together, in vivo data show no effect of α5β1 on erythropoiesis. α4, on the other hand, is required under stress conditions. The contribution of fibronectin-binding was not evaluated, however.

#### 5.5.3. Thrombopoiesis

In vitro, the role of fibronectin on thrombopoiesis was evaluated. While the engagement of α4β1 with fibronectin enhanced megakaryopoiesis, α5β1 seemed less relevant [86]. Even though in vivo studies deleting β1 or β7 in the bone marrow [23] or complete knockout of α4 [52] failed to show an effect on thrombopoiesis, deletion of α4 using Mx-Cre floxed α4, mice and induction of hemolytic stress showed an abnormal response. In normal mice, hemolytic anemia leads to an increase in thrombocytes, because the megakaryocyte-erythrocyte progenitors (MEPs) are shared between erythrocytes and thrombocytes, and hemolysis stimulates erythropoiesis. In Mx-Cre floxed α4 mice, thrombocytes did not increase after hemolytic stress. It is, therefore, conceivable that α4 is involved in these early steps of thrombopoiesis in response to stress [55]. Another possible explanation is that α4β1 expression in stromal cells modulates thrombopoiesis, because in conditions of severe thrombocytopenia, stromal cells produce more thrombopoietin to increase platelet formation [82]. Since Mx-Cre is active in stromal cells, the inability to increase thrombocytes could result from a defect in the stromal cells in the absence of α4.

Fibronectin was implicated in a disease called primary myelofibrosis. This condition is characterized by the accumulation of extracellular matrix in the bone marrow leading to disruption of hematopoiesis, eventually resulting in anemia, thrombocytosis with defective function, leukopenia, and death. Up to 20% of patients develop leukemia [87]. In myelofibrosis, not only is the hematopoietic stem cell defective [87], but mesenchymal stromal cells are also abnormal [88,89]. Fibronectin itself modifies the severity of this disease. Therefore, the experimentally-induced disease is more severe in the presence of EDA-fibronectin [69]. Furthermore, lysyl oxidase and TGF-β, both of which are bound in the matrix in the presence of fibronectin have been implicated in modulating disease severity [15,79,80,81]. Of note, α5β1 integrin interaction with fibronectin mediates the increase in megakaryocytes in an experimental model of myelofibrosis, and counteracting this engagement using an inhibitory antibody against α5β1 diminished the number of megakaryocytes. Taken together, this suggests that the interaction between fibronectin and α5β1, at least in pathologic conditions, plays a role in thrombopoiesis [78].

#### 5.5.4. Lymphopoiesis

Characterization of α4-deficient chimeric mice showed that α4 integrins are not required during fetal life, but are essential for postnatal lymphoid development in the bone marrow [83]. Similarly, β1 integrin was not required for hematopoiesis, but immunization failed to induce IgM production, suggesting that β1 is required for primary T-cell dependent IgM production [64].

Common lymphoid progenitors (CLPs) not only give rise to lymphocytes, but can also differentiate to become dendritic cells once TLRs are activated [84]. This was shown for activation of TLR-9, which normally is an intracellular receptor. However, TLR-9, similarly to TLR-4, signals through myeloid differentiation factor 88 (MyD88) leading to activation of nuclear factor-κB (NF-κB) [90]. For this reason, the involvement of TLR-4 cannot be excluded. Consequently, EDA-fibronectin might also stimulate the differentiation of CLPs. It should be noted for completeness that TLR4 can use a second signaling pathway to activate NF-κB by using the so-called TIR domain-containing adaptor-inducing interferon-β (TRIF) [90], and there is indirect evidence of the involvement of TLR-4 in lymphopoiesis. This could be merely a change in the relationship of progenitors: Whenever myelopoiesis increases in response to EDA fibronectin, for example, there might be a shift away from lymphopoiesis leading to diminished lymphopoiesis relative to the total number of progenitor cells. This inverse relationship can also result from more fundamental cellular mechanisms, such as a change in transcription factors as suggested by the increase in myelopoiesis and decrease in lymphopoiesis in response to upregulation of microRNA 146a [91]. The role of TLRs is, however, most pronounced in later steps of B-cell differentiation, as well as T-cell differentiation in adaptive immunity, where a role for the EDA domain to enhance lymphocytic responses was proposed [92,93,94].

CD44 deletion was associated with an increased proportion of B-cells (and monocytes) in the bone marrow and in peripheral blood, suggesting a possible role in regulating their differentiation [28]. Syndecan-4 is expressed on lymphocytes and monocytes and is involved in chemotaxis and immune cell behavior, but no role in modulating hematopoiesis was elicited yet [43].

## 6. Fibronectin and Malignancy

In malignancy, the expression of fibronectin is associated with a poor prognosis [9]. Indeed, it seems to be part of the premetastatic niche [95]. This is not the case in hematopoietic malignancies. The binding of malignant hematopoietic cells through syndecan-4 to tenascin-C and α4β1 to fibronectin led to their apoptosis. Normal hematopoietic cells, on the other hand, were resistant to syndecan-4 mediated apoptosis, despite the abundance of fibronectin around the normal hematopoietic cells. The authors attributed this to poor expression of syndecan-4 on normal hematopoietic cells, which might explain the absence of defects in hematopoiesis under normal conditions in syndecan-4 knockout mice [85]. Macrophages, however, express the receptor in amounts that are enough to change their cytokine production in response to lipopolysaccharide [42].

## 7. Discussion and Concluding Remarks

The interaction between the hematopoietic stem- and progenitor cells and their niche is multifaceted. Stromal cells of various origins and extracellular matrix components modulate hematopoiesis by affecting migration and homing, attachment and retention, proliferation, as well as differentiation of the stem cells [2,19,59].

One molecule of particular importance is fibronectin, because it is ubiquitous, is relatively large, and consists of various domains that form diverse isoforms [16]. Fibronectin, by binding several different receptors present on the hematopoietic stem cells or the stromal cells, exerts effects that range from modulating homing of stem cells to the last steps of differentiation of immune cell subsets (Figure 1). Indeed, at least in the case of engraftment of stem cells, preculture in the presence of fibronectin can fully replace the supporting ability of stromal cells, highlighting its importance [50].

Even though fibronectin modulates stem cell behavior in vitro, it is better to rely on in vivo data for any conclusions. How fibronectin might affect hematopoietic stem cells in vivo is difficult to address, however, because a total knockout is lethal prenatally, and fibronectin is expressed by almost all cells in the bone marrow, making it available from other sources whenever one source is excluded. Furthermore, whatever the effect of its deletion in specific cell types is, it needs to be pronounced to be detected [19]. Based on the data obtained manipulating fibronectin-binding receptors in vivo, it seems reasonable to conclude that fibronectin, by acting on α4β1 and/or α5β1 on stem cells or on stromal cells, support homing of stem cells to the bone marrow and support retention in the niche, while αvβ3 diminishes proliferation [21,23,25,54,55,59,64].

Direct evidence of the effect of fibronectin on the differentiation of hematopoietic cells is available. EDA-fibronectin originating from the osteoblasts engages the classical fibronectin receptor α5β1 (in a manner that differs from plasma fibronectin lacking EDA) to stimulate myelopoiesis and modulate the immune response towards cancer [19]. These changes were not detected when α5 or β1 were deleted in vivo because the change in myelopoiesis is limited in size, and the change in the immune response was not evaluated. Only a small effect of deletion of α4 was also seen [23,72].

The same holds true for thrombopoiesis and erythropoiesis. Fibronectin exerts a limited effect on thrombocyte formation in physiologic conditions by acting through α4 on the stromal cells [55]. In myelofibrosis, EDA-fibronectin contributes to disease progression by acting on α5β1 [78]. In the case of erythropoiesis, fibronectin stimulation of erythropoiesis only becomes apparent under stress conditions, such as is the case after hemolysis because α4β1 and/or α4β7 are involved [55]. Lymphopoiesis seems unrelated to fibronectin. However, α4β1 is required for later steps of differentiation [64].

The other two potentially relevant fibronectin-binding receptors are CD44, which might facilitate homing and retention, and suppress proliferation [28], as well as TLR-4, which increases stem cell proliferation, their differentiation to myeloid cells, and affects late stages of T- and B-lymphopoiesis [27,60,68,92,93].

Depending on the situation, fibronectin or one of its isoforms might bind another set of receptors. It is possible that during infections, the EDA isoform binds preferably to the hematopoietic stem cell TLR-4 to induce their proliferation and then acts on both α5β1 and TLR-4 to stimulate myelopoiesis. This example highlights the complexity of evaluating hematopoiesis. Indeed, the ubiquitous presence of fibronectin in the bone marrow, together with the wide range of effects it and its isoforms can exert, should be taken into account whenever hematopoiesis is evaluated.

The study of fibronectin’s role in hematopoiesis has been fraught with obstacles, and evaluating the role of fibronectin in the hematopoietic stem cells, lymphopoiesis, and erythropoiesis in vivo is still missing. This is partly due to the large number of cells that produce fibronectin or its isoforms, or compensation in the amount of fibronectin or in the expression of fibronectin receptors with various manipulations [96]. Furthermore, circulating fibronectin can infiltrate various tissues and modulate the local production of fibronectin [9]. Since the complete knockout dies during embryonal development, work focused on using various transgenic models. The availability of mice that do not express the EDA or the EDB isoform has been helpful in evaluating various biological processes [97]. Animals with constitutive expression of the EDA domain were found to support bone marrow fibrosis development compared to mice that fail to produce any EDA-containing fibronectin [69]. With the use of the Cre-loxP system to delete fibronectin in defined cell populations, it was possible to characterize the role of fibronectin originating from osteoblasts in myeloid cell differentiation [19]. More of such exciting findings are expected as the constituents of the hematopoietic stem cell niche are being characterized, allowing specific deletion of fibronectin in these cell populations.

Because of the critical role of hematopoiesis for survival, it would have been naïve to expect that deletion of a single molecule would throw hematopoiesis off their tracks. Instead, the system is designed such that the loss of one molecule can be compensated for with several mechanisms cooperating for an optimal response of hematopoiesis to the various challenges. Many questions still need to be answered and will keep those evaluating the role of extracellular matrix in hematopoiesis busy for years to come.

## Figures and Tables

**Table 1:** Fibronectin can engage a large number of receptors—only receptors that were shown to be expressed on hematopoietic stem cells are listed.

Fibronectin Receptors Expressed on Hematopoietic Stem Cells	
Integrins	α5β1 [21]
	α4β1 [22]
	α4β7 [23]
	α9β1 [24]
	αvβ3 [25]
TLR-4	[26,27]
CD44	[28]

**Table 2:** Fibronectin supports hematopoietic stem cells in migration and homing, retention, and proliferation by acting on various receptors. Evidence for fibronectin involvement was established in vitro. Because fibronectin deletion in mice leads to embryonal death, the role of fibronectin in these steps in vivo was implied by evaluating the various receptors as outlined.

Function of Hematopoietic Stem Cell	Role of Fibronectin	Role of Fibronectin Receptors
Migration and homing	Fibronectin acts in vitro to maintain the ability of stem cells to engraft [50]	Migration diminishes if β1 is deleted in hematopoietic cells [47,51]
		α4β1 and α5β1 are required for homing in adults [21,53,54,55]
		CD44 supports homing [28,56]
Retention	Fibronectin supports attachment in vitro [57]	α4β1 enhances retention through actions on stromal cells [55,58]
		αvβ3 maintains long-term repopulation capacity, requires thrombopoietin [25]
		CD44 stimulates adhesion. Possible cooperation with α4β1 [38]
Proliferation	Fibronectin stimulates cell proliferation [9]	αvβ3 engagement suppresses proliferation [25,59]
		TLR-4 activation with LPS increases proliferation [60]
		CD44 inhibits proliferation [28]

**Table 3:** Fibronectin supports myelopoiesis and contributes to pathologic thrombopoiesis (in myelofibrosis). It exerts a limited role in erythropoiesis or lymphopoiesis.

Differentiation Step	Role of Fibronectin	Role of Fibronectin Receptors
Myelopoiesis	EDA fibronectin stimulates myelopoiesis and changes the immune response towards cancer [19]	CML is associated with abnormal adhesion to α4β1 and α5β1 [71]
		α4 deletion leads to inefficient myelopoiesis [52,72]
		α5, αv, β1, and β7 deletion do not affect myelopoiesis [23,72]
		TLR-4 boosts myelopoiesis [66,70,73,74]
		CD44 knockout increases monocytes in the bone marrow and circulation [28]
Erythropoiesis	Fibronectin stimulates erythropoiesis in vitro [75,76]	α5 deletion did not affect erythropoiesis [77]
		α4 deletion or combined β1 and β7 deletion showed limited recovery after hemolysis [23,55]
Thrombopoiesis	In myelofibrosis, fibronectin contributes to the disease directly, by binding to α5β1 with the possible involvement of stored molecules [15,69,78,79,80,81]	α4, β1, and β7 deletion did not affect thrombopoiesis [23,52]
		α4 deletion in hematopoietic and stromal cells simultaneously prevented the expected increase after hemolysis [55,82]
Lymphopoiesis		α4 is required for postnatal lymphopoiesis [83]
		β1 is required for T-cell dependent IgM response to immunization [64]
		TLR-4 affects B- and T-cell maturation [84]
		CD44 deletion increases B-lymphocytes [28]
		Syndecan-4 modulates chemotaxis [43]
